# Prevalence of *Schistosoma intercalatum* and *S. haematobium* Infection among Primary Schoolchildren in Capital Areas of Democratic Republic Of São Tomé and Príncipe, West Africa

**Published:** 2012

**Authors:** TB Chu, CW Liao, YC Huang, YT Chang, ASRJ Costa, DD Ji, T Nara, A Tsubouchi, Peter WS Chang, WT Chiu, CK Fan

**Affiliations:** 1 School of Health Care Administration, College of Public Health & Nutrition, Taipei Medical University, Taipei, Taiwan; 2 Dept. of Parasitology, College of Medicine, Taipei Medical University, Taipei, Taiwan; 3 Dept. of Molecular and Cellular Parasitology, Juntendo University School of Medicine, Tokyo, Japan; 4 Taiwan Medical Mission in São Tomé, Democratic Republic of São Tomé and Príncipe; 5 Ministry of Health and Social Affairs, Democratic Republic of São Tomé and Príncipe; 6 Laboratory of Parasitic Diseases, Center for Diseases Control, Dept. of Health, Taipei, Taiwan; 7 School of Public Health, College of Public Health & Nutrition, Taipei Medical University, Taipei, Taiwan; 8 Dept. of Neurosurgery, School of Medicine, Taipei Medical University, Taipei, Taiwan; 9 Center for International Tropical Medicine, Taipei Medical University, Taipei, Taiwan; 10 Graduate Institute of Medical Sciences, College of Medicine, Taipei Medical University, Taipei, Taiwan

**Keywords:** Schistosomiasis, Schoolchildren, Democratic Republic of São Tomé and Príncipe, West Africa

## Abstract

**Background:**

A parasitological survey of *Schistosoma haematobium* and *S. intercalatum* infection among primary schoolchildren in capital area of Democratic Republic of São Tomé and Príncipe (DRSTP) was undertaken.

**Methods:**

Subjects with positive infection were confirmed by the detection of *S. haematobium* ova in the urine or *S. intercalatum* ova in the stool by using centrifugation concentration or merthiolate-iodine-formalin concentration method. Totally, 252 urine and stool samples, respectively, were obtained from apparently healthy schoolchildren, of which 121 from boys (9.8 ± 1.4 yr) and 131 from girls (9.7 ± 1.3 yr).

**Results:**

None of participating schoolchildren were found having *S. haematobium* ova in the urinary specimen. While, among 4 primary schools studied, only schoolchildren from Saint Marçal were detected with *S. intercalatum* ova in the fecal specimen, making the overall prevalence of *S. intercalatum* infection among schoolchildren was 2.4% (6/252) and girls had insignificantly higher prevalence (3.1%, 4/131) than that (1.7%, 2/121) in boys (χ^2^ = 0.5, *P* = 0.5).

**Conclusion:**

Water control and sanitation as well as snails eliminated by molluscicides are urgently needed to reduce *S. intercalatum* infection in DRSTP inhabitants.

## Introduction

Chronic schistosomiasis, one of the major health problems in tropical and sub-tropical countries, affects more than 200 million people worldwide, and the majority of cases occur in Sub-Sahara Africa (SSA) (1). Although most of the schistosomiasis cases are due to *Schistosoma haematobium* infection (2), some minor Schistosomal species such as *S. intercalatum* infection reportedly is limited to some western and central African countries (3).

*Schistosoma haematobium* inhabits the vesical plexus of veins around the human bladder and urinary tract and only occasionally the veins of the rectum and portal systems. The adult female lays eggs present in urine that are 83 to 187 by 60 µm and that are characterized by a terminal spine; while *S. intercalatum* inhabits the mesenteric and portal venous systems of its human host and the adult female lays eggs present in stool that are 140 to 240 by 50 to 85 µm and that are characterized by a long terminal spine. Nonetheless, the eggs of *S. haematobium* and *S. intercalatum* are practically indistinguishable (4). Possible consequences of *S. haematobium* infection include haematuria, dysuria, nutritional deficiencies, lesions of the bladder, kidney failure, an elevated risk of bladder cancer and in children-growth retardation (2); while *S. intercalatum* shows a comparative mild pathogenicity that causes rectal schistosomiasis characterized by a low location of the lesions at the level of the rectum and sigmoid colon and relatively minor liver pathology (3).

*Schistosoma intercalatum* instead of *S. haematobium* has been reportedly the only schistosomal species endemic in the Democratic Republic of São Tomé and Príncipe (DRSTP) for nearly two decades, particularly of São Tomé Island (5–7). Previous studies indicated the average infection rate of *S. intercalatum* among schoolchildren during 1994-2005 showed a range of 11.7% to 36.2% (5–7). Nevertheless, controversial exists that Pampiglione et al. (1987) also indicated the *S. haematobium* infection could be found among inhabitants in the DRSTP (8).

Primary schoolchildren are particularly vulnerable to schistosomiasis due to their habits of playing in the water and hence they are the ideal target group to investigate the schistosomiasis prevalence and the data collected from this age group thus can be used to assess not only whether schistosomiasis threatens the health of school- age children, but also as a reference for evaluating the need for community intervention (9).

The present study intended to investigate the infection status of schistosomiasis as well as to monitor whether *S. intercalatum* and *S. haematobium* co-exists in this country by detection of ova from urinary and fecal specimen from schoolchildren in Capital areas of Agua-Grande Province in DRSTP.

## Materials and Methods

### Geographical description, study population, and subject selection

**Fig. 1:**
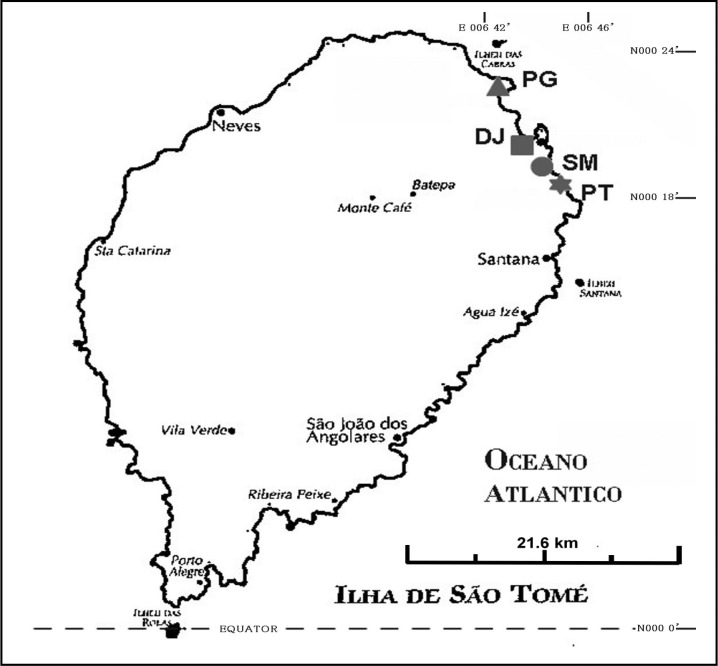
Map indicating the geographic location of 4 primary schools (Saint Marçal, SM; Pantufo, PT; Praia Gamboa, PG; and 1 de Junho, DJ) located in capital areas of Democratic Republic of São Tomé and Príncipe, Western Africa

The DRSTP consists of two islands of Sao Tome and Principe and a number of smaller islets in the Gulf of Guinea. Sao Tome lies approximately 180 miles from Gabon on the West African coast and is crossed by the equator at its southern tip. The climate is tropical with two rainy seasons. The total number of inhabitants in the DRSTP is estimated to be 160000, and the total number of inhabitants in Sao Tome Island is approximately 150000. This study started from 6∼24, October of 2010. Schoolchildren of grade 4∼5 (mean age ± SD: 9.8 ± 1.3 yrs) from 4 primary schools (Saint Marçal, Pantufo, Praia Gamboa, and 1 de Junho) located in Capital areas (Fig. 1) having not enrolled in the deworming programme in 2010 were selected to participate in the present study according to the suggestions by Ministry of Health & Social Welfare after informed consent was obtained from their parents or guardians or school representatives.

### Urine sample collection and performing procedure

A single terminal urine sample was collected from each participant between 10.00 and 14.00 hours, reportedly the maximum ova excretion occurs (10). In total, 252 urine samples were obtained from apparently healthy schoolchildren, of which 121 from boys and 131 from girls. The mean ages were similar in both genders (boy: 9.8 ± 1.4 yrs *vs* girl: 9.7 ± 1.3 yrs). Briefly, ten milliliters of each of the well-mixed urine samples was poured into a quantitative centrifuge tube specific for urinary cells/parasites counting (cat. no. ParaQ 6b, High Skill Co., Ltd., Chupei, Taiwan) centrifuged at 2000 rpm for 3 min. The supernatant was discarded but about 0.6 ml residual urines were still retained in the bottom of tube and then 50 µl of the urinary solution was dropped into a counting chamber (cat. no. ParaQ 5b); thereafter the number of *S. haematobium* ova present in the chamber under the microscope at 100 x magnification was calculated, finally the number will be multiplied by 12 to represent a total number of ova present in 10 ml urine specimen (11). The mean number of ova per 10 ml urine present in all of positive samples was defined as geometric mean intensity (GMI), and any samples that contained less than 50 ova /10 ml was regarded as light infection; however the figure was equal to or more than 50 ova /10 ml were regarded as heavy infection as suggested by World Health Organization (12).

### Stool sample collection and performing procedure

We distributed the stool containers (cat. no. ParaQ1, High Skill Inn., Taipei, Taiwan) along with a data sheet containing personal information e.g., school, grade, name, gender, and age, to the participating schoolchildren and asked them to defecate and bring stool container back to us in the school on next day morning. In total, 252 stool samples were obtained from apparently healthy schoolchildren, of which 121 from boys and 131 from girls. After we recovered the stool containers, the individual fresh fecal specimen were further processed within 4 hours and examined presence of any parasites by using merthiolate-iodine-formalin (MIF) concentration method (13). For examination of any parasite in fecal materials, a 5 ml of MIF solution containing a 4.7-ml merthiolate-formaldehyde solution and a 0.3-mL iodine solution (cat. no. ParaQ3, High Skill Inn.) were added into each fecal container with stool sample for at least 4 hrs. The MIF-stool sample was then filtered through a layer of gauze in the bottom of the fecal container, and the eluted solution in a collection tube (cat. no. ParaQ2, High Skill Inn.) was centrifuged at 2,000 rpm for 2 minutes. The supernant fluid was then discarded and the residual pellet was examined for helminth ova and protozoan cysts/trophozoites under a microscope (Olympus BX41, Tokyo, Japan).

### Ethical approval

Ethical approval for the study was obtained from the Ministry of Health & Social Affairs of the DRSTP.

### Statistical analysis

Statistical analysis was performed using SPSS software system (SPSS Inc., Chicago, IL, USA). Chi-square test (χ^2^) was calculated and when *P* values less than 0.05 were considered to be statistically significant.

## Results

None of participating schoolchildren were found having *S. haematobium* ova in the urinary specimen, thus the overall infection rate was 0.0% (0/252) (Table 1). While, among 4 primary schools studied, only schoolchildren from Saint Marçal were detected positive for *S. intercalatum* ova in the fecal specimen, thus the infection rate was 8.2% (6/73). Altogether, the overall prevalence of *S. intercalatum* infection in present study was 2.4% (6/252) and girls had insignificantly higher prevalence (3.1%, 4/131) than that (1.7%, 2/121) in boys (χ^2^ = 0.5, *P* = 0.5) (Table 1).

**Table 1:** Prevalence of *Schistosoma intercalatum* and *S. haematobium* infection among schoolchildren in Capital areas of Democratic Republic of São Tomé and Príncipe, Western Africa

Variable	Mean (S.D) age (Yr)	No. of examined	*S. intercalatum*		*S. haematobium*	
			No. of positive	% of positive	No. of positive	% of positive
**School**						
Saint Marçal	9.9 ± 1.2	73	6	8.2	0.0	0.0
Pantufo	10.4 ± 1.3	74	0	0	0.0	0.0
Praia Gamboa	8.8 ± 1.4	39	0	0	0.0	0.0
1 de Junho	9.5 ± 1.1	66	0	0	0.0	0.0
						
**Gender**						
boy	9.8 ± 1.4	121	2	1.7	0.0	0.0
girl	9.7 ± 1.3	131	4	3.1	0.0	0.0
**Total**	9.8 ± 1.3	252	6	2.4	0.0	0.0

## Discussion

Although Pampiglione et al. (1987) indicated the *S. haematobium* infection could be found among inhabitants in the DRSTP (8), however, we were unable to detect any *S. haematobium*-infected schoolchildren in the present study. Our results re-confirmed that *S. haematobium* did not seem endemic in DRSTP and also present finding is very similar to previous studies indicating no *S. haematobium* egg was found in 782 and 181 urine specimens from the local population and schoolchildren, respectively in DRSTP (5, 7).

In contrast, *S. intercalatum-*infected schoolchildren could be found in present study and the overall infection rate of *S. intercalatum* among those schoolchildren was not high, reaching nearly 2.4%. This figure is much lower than 36.2% in a previous study conducted on schoolchildren in DRSTP in 2005 (7). Interestingly, among the 4 primary schools studied, only Saint Marçal was detected to be present with *S. intercalatum-*infected schoolchildren. It is postulated due to there is a river close to this school that increases the opportunity for schoolchildren to expose themselves to the contaminated river, in addition, most of the schoolchildren from Saint Marçal were reported lacks of house sanitation facility thus they often take a bath in this river as resulting in higher contact with infective water bodies (7). However, it remains necessary to eliminate the transmitting snails so as to block the infective cercariae thus decreasing this parasite infection to inhabitants living near this district.

Present study also found girls had higher infection rate than that in boys. It may be explained by that they often accompany with their mothers to undertake water- related activities e.g., swim and bathe thus resulting in higher exposure opportunity to *S. intercalatum* infection. Other factors that may account for the presence of *S. intercalatum*-infected schoolchildren include the absence of a community-based control programme against schistosomiasis and probably the absence of mass or targeted health education in most Africa countries including DRSTP due to poor financial resource (14). Moreover, although DRSTP has undertaken schoolchildren based deworming programme by using mebendazole alone regimen annually since 2005, it is merely useful in killing some soil-transmitted helminths e.g., *Ascaris lumbricoides* but not *S. intercalatum* (15).

Although we did not investigate on the snail host in the river close to the school, the snail host of *Bulinus forskali* for *S. intercalatum* can be found in slowing-moving river (16), and since its warmer temperature (27°C) suitable for parasite development in the snails in the river, it seemed likely that inhabitants particularly children and women are highly susceptible to *S. intercalatum* infection through contact with water contaminated by cercariae thus leading to increased opportunity of newly or repeatedly acquired *S. intercalatum* infection in the district which Saint Marçal is located in. Considering individuals infected with *S. intercalatum* may suffer from rectal schistosomiasis characterized by a low location of the lesions at the level of the rectum and sigmoid colon and relatively minor liver pathology (3), if they are not treated properly, such mild complications may still lead to severe consequence (17).

Altogether, the present report will be useful in planning an integrated schistosomiasis control programme in the neglected country and it is also recommended that in DRSTP not only everybody should be dosed with praziquantel, irrespective of age; but also water control and sanitation as well as snails eliminated by molluscicides are urgently needed to reduce *S. intercalatum* infection to DRSTP inhabitants.
